# Study protocol: Home-based physical rehabilitation for survivors of a critical illness [ACTRN12605000166673]

**DOI:** 10.1186/cc4949

**Published:** 2006-06-22

**Authors:** Doug Elliott, Sharon McKinley, Jennifer A Alison, Leanne M Aitken, Madeleine T King

**Affiliations:** 1 Faculty of Nursing, Midwifery and Health, University of Technology, Sydney, New South Wales, Australia; 2 Critical Care Professorial Unit, University of Technology, Sydney & Northern Sydney Central Coast Area Health Service, New South Wales, Australia; 3 School of Physiotherapy, Faculty of Health Sciences, The University of Sydney, New South Wales, Australia; 4 Princess Alexandra Hospital & Griffith University, Queensland, Australia; 5 Centre for Health Economics, Research & Evaluation, University of Technology, Sydney, New South Wales, Australia

## Abstract

**Introduction:**

Numerous primary studies and several review papers have highlighted delayed physical and psychological recovery for survivors of critical illness, often beyond 6 months after discharge. This randomized controlled trial with blinded assessment aims to test the effects of an 8-week, home-based, individually tailored physical rehabilitation programme on physical and psychological recovery for survivors of a critical illness after discharge from hospital.

**Method:**

Participants are survivors of a critical illness discharged from nine intensive care units (ICUs) in Australia, who are aged 18 years or older, in an ICU longer than 48 hours, discharged home to self-care or carer (non-institutional care), able to participate in physical rehabilitation, and within the hospitals' local geographical areas for home visits. The study is based in participants' home environments. Blinded assessments at weeks 1, 8 and 26 after hospital discharge examine physical functioning, exercise capacity, health-related quality of life and psychological well being. The intervention is graded, individualized endurance and strength training prescribed by a pulmonary rehabilitation physiotherapist over an 8-week period, with three home visits, five follow-up phone calls, and a printed exercise manual supporting the training. Initial focus is on lower limb exercises and walking, with warm-up stretches, and progresses to the addition of core stabilization and upper limb exercises.

**Results:**

The burden of a critical illness is well documented. This novel study will determine whether a home-based physical rehabilitation programme improves the recovery trajectory for survivors of critical illness. The projected sample size of 200 patients aims to detect a clinically important 10% improvement in physical functioning. The study will also examine whether other important physical and psychological measures are improved.

**Conclusion:**

This multicentre, randomized controlled trial will examine outcomes that are meaningful to patients, their family and society, namely functional ability and well being. The study will also target a health problem that is likely to increase as the population ages. If the programme is effective, it will provide a model that can be easily adapted and adopted by existing primary care or community services to improve the recovery of individuals following critical illness.

## Introduction

Examination of patient outcomes following a critical illness, over and above survival, has become an important topic of critical care research, particularly over the last decade [1,2]. Although continuing patient follow up by the intensive care unit (ICU) team following ICU discharge is not a traditional routine practice, this lack of continuity is now being questioned [3]. The development of critical care outreach or liaison teams is evident, particularly in the UK [4] and to a lesser extent in Australia [5], with a focus on early detection of clinical deterioration. Importantly, however, systematic follow up with physical rehabilitation interventions for survivors of critical illness with physical debilitation is not routinely provided, particularly after hospital discharge, although there are exceptions [6].

A critical illness requiring admission to a general ICU affects over 130,000 adult Australians per year [7]. Although survival rates approximate 86% [7], functional recovery for individuals is often delayed beyond 6 months after hospital discharge [8-10]. There is commonly physical de-conditioning [11] as well as psychological sequelae [12,13], adding to the burden of illness for our society [3]. A number of review papers identified a plethora of observational studies that confirm this delayed recovery [8-10]. The methods and measuring instruments used to explore patient outcomes have evolved from crude mortality and morbidity indicators to more patient-centred concepts such as functional status and health-related quality of life (HRQOL) [9]. Some methodological limitations with these more recent studies remain, including the lack of randomized trials, use of unvalidated measuring instruments and lack of control of extraneous variables [14]. Despite these limitations, it is clear that significant sequelae exist for a substantial proportion of critical illness survivors. However, there is a lack of evidence for the benefit of any specific service provision for recovering survivors from critical illness [3,15], with only a few published interventional studies available [6,16]. None have tested the effect of home-based rehabilitation on patient recovery.

We propose that a focused home-based approach to physical rehabilitation, in addition to usual community-based health services, will improve the HRQOL and recovery of individuals surviving a critical illness. The rehabilitation programme for this cohort reflects similar successful programmes applied in patients with cardiac and respiratory disease [17,18] by optimizing functional recovery, particularly during the first few months after such an illness. However, there is a dearth of literature examining similar programmes involving the general critical care population [3,6,15].

## Materials and methods

This multicentre randomized controlled trial (RCT) tests the effect on physical and psychological function of an eight-week home-based rehabilitation programme for individuals who have survived a critical illness. Physical function is measured using the Physical Functioning (PF) subscale of the Short-Form-36 version 2 (SF-36) Health Survey [19] and the 6 minute walk test (6 MWT) [20]; psychological function is measured using the Depression Anxiety and Stress Scale (DASS-21) [21] and the Impact of Events Scale (IES) [22]. Other aspects of HRQOL are measured using the SF-36. The recruitment frame for the study currently includes three tertiary referral hospitals and three metropolitan hospitals in Sydney, New South Wales, and two tertiary referral hospitals and a metropolitan hospital in Brisbane, Queensland, Australia.

### Research hypothesis and aims

The primary research hypothesis is that survivors of a critical illness who participate in the physical rehabilitation programme will have better physical function, as measured by a difference of 10 points on the SF-36 PF subscale, when compared with those who receive usual care at eight weeks after hospital discharge (short-term effect), which will persist at 26 weeks (long-term effect).

The secondary aims of the study test the effectiveness of the rehabilitation programme in terms of the following: improvement in other domains of HRQOL, as measured using the SF-36 [19]; better physical endurance, as measured by the 6 MWT [20]; improved psychological recovery, as measured using the DASS-21 [21]; less psychological distress following critical illness, as measured using the IES [22]; and reduced use of health services.

We do not propose to test *a priori *hypotheses about these secondary outcomes, because there is insufficient evidence available about the effect of the intervention on these aspects of health and well-being in this patient population and context. Rather, the study will yield information useful for formulating future hypotheses about these secondary outcomes.

### Sample size

Sample size was calculated for the SF-36 PF subscale for a two-sided hypothesis test with a type I error rate of 0.05 and a type II error rate of 0.20 (80% power). The clinically important difference and the standard deviation estimates used in our sample size calculations were based on our pilot data [23,24] and reports for similar cohorts and contexts [6,19,25,26]. At baseline we anticipate that both groups will have mean PF scores of 45. We postulate that the control group will improve by 5 points at 8 weeks, with the intervention group improving by 15 points, giving a difference of 10 points between the two study groups. Using the 10 items that comprise the PF subscale of SF-36, an improvement of 15% represents a change from 'limited a lot' to 'limited a little' on three items in the scale, for example in climbing stairs or walking particular distances. These changes reflect significant clinical improvement in physical function [27].

A sample of 100 patients per study group is required to detect this difference, assuming similar group variance (standard deviation = 25) [23,24]. We will over-enrol by 20% to account for losses to follow-up (10% study attrition [6]; 10% mortality at 6 months after hospital discharge following a critical illness [7,28]). Reasons for loss to follow up will be recorded (such as, death, withdrawal). We will minimize avoidable loss to follow up at 6 months by maintaining monthly phone contact with all participants during the study period. We will therefore enrol up to 240 participants across the recruitment sites over an 18-month recruitment period.

### Procedure

**Figure 1:**
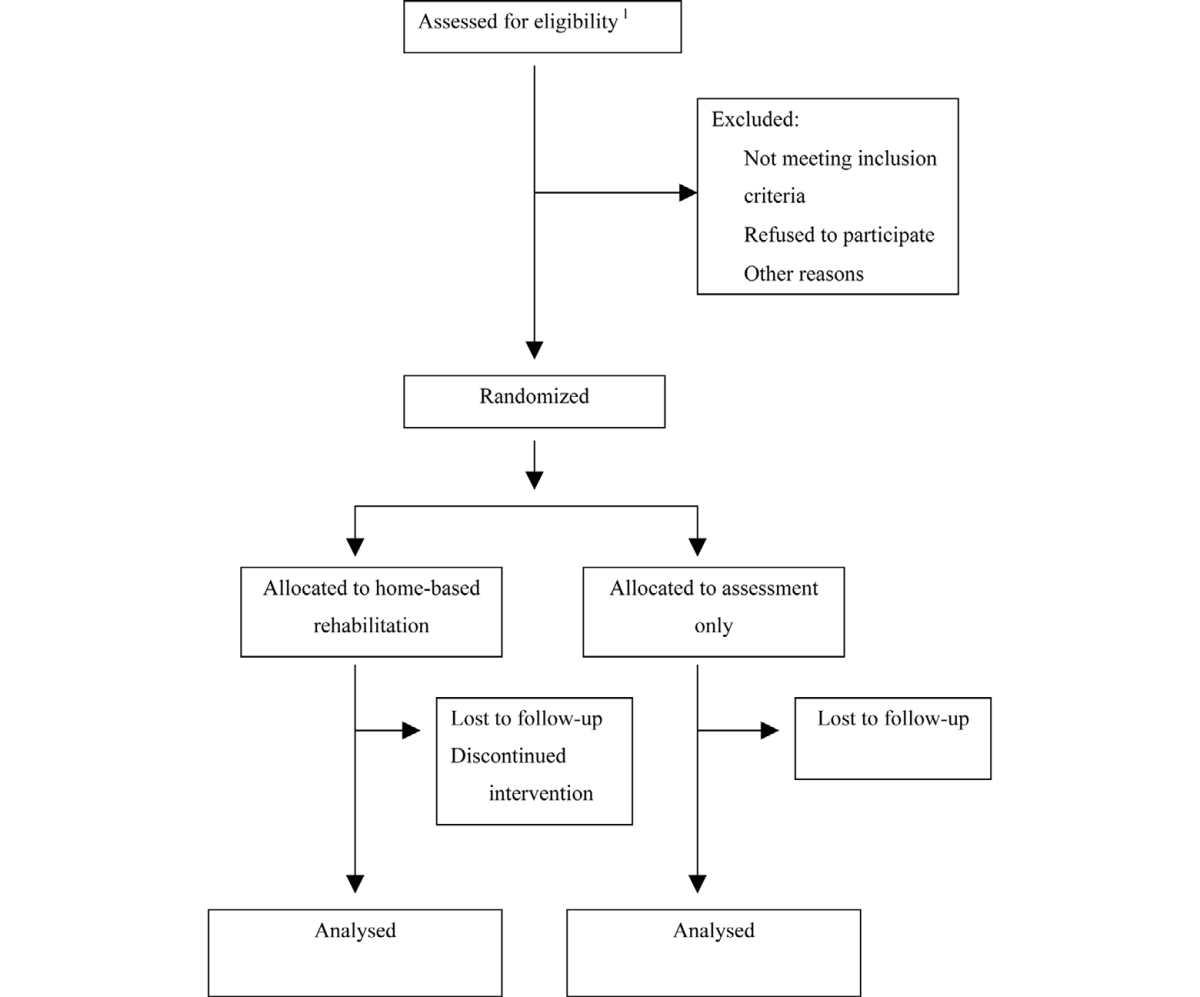
**Participant enrolment and retention flow diagram**. ^1^The inclusion criteria are as follows: age ≥18 years or older; intensive care unit length of stay >48 hours; receipt of mechanical ventilation for ≥24 hours; discharged home to self-care or carer; residence within the hospitals' local catchment areas to enable home visits; no neurological, spinal or skeletal dysfunction preventing participation in physical rehabilitation; not receiving palliative care; and no organised rehabilitation related to ongoing chronic disease.

Approval to conduct the study was obtained from the Human Research Ethics Committees of the hospitals acting as recruitment sites and the universities of the investigators. The study protocol is registered with the Australian Clinical Trials Registry (ACTRN12605000166673) [29]. The planned flow of patients through the study reflects recommendations from the CONSORT (Consolidated Standards of Reporting Trials) statement [30], and is illustrated in Figure 1.

#### Inclusion criteria

To be eligible for enrolment, participants must be aged 18 years of age or older; have an ICU length of stay greater than 48 hours; have received mechanical ventilation for 24 hours or longer; be discharged home to self-care or carer (non-institutional care); reside within the hospitals' local geographical areas to enable home visits (approximately 30 km radius); have no neurological, spinal, or skeletal dysfunction preventing participation in physical rehabilitation; not be receiving palliative care; and have no organized rehabilitation related to ongoing chronic disease management (such as, pulmonary rehabilitation, cardiac rehabilitation).

#### Recruitment and randomization

Eligible patients are approached immediately before or after ICU discharge, and informed voluntary consent obtained. After participant consent, the site project officer contacts an independent telephone randomization service for the participant study number and group allocation. The service uses the blocked random allocation sequences (one for each recruitment site) generated using SAS software (SAS Institute Inc., Cary, NC, USA) by the study statistician (MTK), who is the only member of the study team with prospective knowledge of these sequences. Effectiveness of the randomization process will be examined by an independent Data Monitoring Committee (see Data management and analysis, below).

#### Assessment

All participants are assessed at home within 1 week of hospital discharge by an assessor who is blinded to group assignment (Table 1). The control group receives usual care after hospital discharge. The three assessment visits for the control group are additional to 'usual care'. Although this contact is unavoidable and may have a placebo effect, any effect will reduce the apparent effectiveness of the intervention in this RCT. The treatment effect is therefore measured relative to the control group in the study, although in reality the comparator would be usual care without assessment contact.

Following the first assessor visit, participants allocated to the intervention group are visited at home by a dedicated trainer in weeks 1, 3 and 6 to provide individualized verbal and written instructions on their planned exercise programme. Exercise participants are telephoned by the trainer in weeks 2, 4, 5 and 7 to monitor their progress. Home-based follow-up assessments occur at 8 and 26 weeks after discharge for all participants (Table 1).

#### Researcher safety protocol

A safety protocol is used to ensure assessor and trainer safety during home visits: a list of visits is held by the responsible investigator for each recruitment site with the participant's address, date, time and approximate duration of each visit; the assessor/trainer carry a mobile phone for contact; the assessor/trainer sends a text message to the investigator before entering the home and when the visit is complete; and if no contact is made, the investigator phones the assessor/trainer and then the participant's home phone (if connected).

### Outcome measures

The primary outcome measure is the physical functioning of study participants. We selected the SF-36 PF subscale as the corresponding measuring instrument [19] because it has demonstrated reliability, validity and responsiveness in the post-ICU population [31], and is the instrument most commonly used to assess health status in this patient cohort [3,8-10]; this will allow comparison between this study and similar cohorts.

The secondary outcome measures are as follows: exercise capacity; HRQOL; and constructs of psychological well being. Exercise capacity is measured using the 6 MWT [20]. The 6 MWT is performed twice at each assessment to account for any learning effect, with the best result recorded for analysis. During the 6 MWT, participants are monitored continuously using a portable pulse oximeter (measuring pulse rate and oxygen saturation), with their exertion level assessed and documented during the test [20] using the Borg perceived exertion scale [32]. HRQOL is assessed using other subscales of the SF-36 [19], including effect of Physical Health on Role (Role – Physical), Bodily Pain, General Health, Vitality, Social Functioning, effect of Emotional Health on Role (Role – Emotional), and Mental Health.

We also measure constructs of psychological well being as important aspects of outcome in this patient group [3,15]. Previous studies have demonstrated that psychological distress (anxiety, depression, worry) during recovery correlate with an increased incidence of symptoms consistent with post-traumatic stress [6], although any causal relationships between critical illness, physical recovery and psychological distress are not yet clear. Psychological state is assessed using DASS-21 [21] and the IES [22]. The DASS-21 is a psychometrically robust measure of affect, using 21 items to examine the three psychological dimensions of depression, anxiety and stress, described by physical symptoms, cognitions or feelings, and rated on a 4-point scale over the preceding week. Australian normative data are available for comparison. The IES uses 15 items to measure avoidance behaviour and intrusive thoughts associated with psychological distress consistent with symptoms of post-traumatic stress disorder.

In addition, participants record all their contacts with health services during the study period, including the number and purpose of visits to their family doctor, medical specialist, community nurse, pharmacist, community health centre, or emergency department, or hospitalization since the index hospital episode or previous assessment. The battery of questionnaires takes 20–30 minutes to complete.

### Intervention

Participants randomized to the intervention group receive an eight-week, home-based physical rehabilitation programme that focuses on strength training and walking. All exercises use standard approaches for improving muscle strength and endurance within a rehabilitation setting [33,34]. Participants are visited at home in week 1 for exercise prescription and supervised training. Physical rehabilitation training involves graded, individualized endurance and strength training prescribed by a pulmonary rehabilitation physiotherapist. Training initially focuses on lower limb exercises and walking, with warm-up stretches. As participants progress, core stabilization and upper limb exercises are introduced. Participants are also visited by the trainer in weeks 3 and 6 for assessment of progress and reinforcement of the exercise programme, and are contacted by the trainer by telephone in nonvisit weeks (2, 4, 5, 7 and 8) to reinforce the exercise programme.

#### Exercise manual

An illustrated exercise manual supports the participant's training and graded progression. The manual contains three parts: 'how hard should you exercise', which details the modified Borg Scale and provides information about participant safety; the exercise program; and how to progress the exercises. The exercise program details exercises in five components: endurance exercise (walking), lower and upper limb strengthening, core stabilization, flexibility, and stretches. A total of 16 different exercises are numbered, named, illustrated and described, to facilitate participant-trainer communication and exercise progression. This includes four stretching, three flexion, and three core stabilization exercises, which are included in the exercise prescription based on assessment of the participant's capabilities and needs by the exercise trainer.

#### Strength training

Assessment of strength uses an eight-repetition maximum (8 RM) protocol (that is to say the exercise or weight that can only be completed eight times in one set). An 8 RM protocol is less likely to cause undue strain for the participant than other assessments of strength that require heavier weights. Strength training includes upper (biceps, triceps, shoulder abductors/adductors) and lower limb (quadriceps, hamstrings, hip abductors and extensors) muscle groups. The initial strength training prescription is one set of 8 RM for each activity, progressing to three sets. Further progression is based on increasing weight (0.25–1.5 kg for arm exercises using food cans or bags of rice, or increasing the step height or weight for lower limb exercises), with levels of progression described.

#### Endurance training

Exercise prescription for endurance training is based on the results of the 6 MWT during the baseline assessment visit. Training intensity commences at 80% of peak walking speed. Extra activities are prescribed based on a level of perceived exertion of moderate intensity (3–4, modified Borg scale). The programme uses a walk-rest-walk approach, with the duration of walking varying according to the participant's ability and condition; 12 levels of walking are described ranging from 1 to 60 minutes. Participants work toward an optimal goal of training on 5 days per week for 20–30 minutes by the end of the programme.

### Data management and analysis

Data are entered into a purpose-built Access database at the three coordinating sites; monthly site reports on enrolment, randomization and participant follow up are submitted to one central site and monthly summaries of the information shown in Figure 1 for the whole study are given to all investigators. Analysis is by intention to treat. Analysis of covariance of the PF subscale scores for the control and intervention groups at 8 weeks will be conducted, using the baseline measure as a covariate. Multivariate regression techniques will describe the effect of the intervention on the secondary outcome measures (other SF-36 domains, 6 MWT, DASS subscales and IES subscales), and correlations among all outcome measures will be estimated. Baseline characteristics of patients lost to follow up at 8 weeks and 26 weeks will be compared with patients who completed follow up to assess patterns of loss to follow up and provide insights into generalizability of the results. A Data Monitoring Committee independent of the research team will be convened to review study progress and interim and final analyses [35].

### Pilot study

The intervention and all study procedures were tested in a small dual-site pilot RCT before commencement of the fully powered study described here. The pilot study demonstrated the feasibility of the training programme and study procedures (recruitment, retention, outcome measures), and enabled refinement of the training manual, researcher safety procedures, protocol implementation across multiple sites, and data collection and management strategies.

## Results and discussion

The burden for survivors of a critical illness has been well documented in many observational studies, in which the recovery trajectory is often prolonged and suboptimal. However, intervention studies with this clinical cohort are less common. To our knowledge, the proposed research is the first study internationally to use a home-based rehabilitation programme in this patient group. An individualized, home-based programme negates the need to attend an outpatient clinic located in a hospital on a regular basis. This is particularly important for individuals who reside in regional or rural areas but were treated in a metropolitan ICU, as well as those who choose not to or are unable to participate in hospital-based programmes for other reasons such as lack of mobility or transport. The provision of such a programme through local community health services would allow survivors of a critical illness to engage in the programme regardless of place of residence and other mobility and access constraints.

The significance of this research can be considered from a number of aspects. The first aspect pertains to the patient and their family. The effects of a critical illness can be devastating on all aspects of HRQOL, including physical, psychological, social, fiscal and spiritual. This intervention aims to improve the recovery for patients to their optimal level of functioning following a critical illness using a structured programme of physical exercise. The second aspect concerns health professionals. Following the patient through their recovery process will help to inform clinicians regarding the appropriateness and long-term impact of ICU management. Development of outreach services to ensure any benefits gained are not lost by insufficient support after intensive care is an important component of clinical management within an extended episode of care. Finally, the research findings will be of value to health service managers because they may facilitate utilization of health care resources appropriately and efficiently to improve patient outcomes for survivors of a critical illness.

If the study findings are positive and conclusive, this approach could be adopted by any community or primary health service in both metropolitan and rural settings, given appropriate training and support. The exercise manual uses standard exercises and training patterns. Facilitation and monitoring of individuals engaged in the programme could be undertaken by a variety of health professionals within primary care or community health services (for example, general practitioners, community nurses, practice nurses, physiotherapists) after some initial training and with the aid of resources such as the training manual.

The innovative aspects of the project are in their application to this important but often heterogeneous group of patients and in the project's home-based focus, which frees patients from the need to attend hospital outpatient clinics. This research will also inform clinicians and health service providers about one approach within a range of outreach services that best serve the needs of patients following critical illness and lead to better long-term patient outcomes with appropriate utilization of scarce resources.

## Conclusion

This study uses a multicentre RCT design to provide the best evidence to examine the effectiveness of a novel application of physical rehabilitation practices to an important clinical cohort. The project's significance relates to two key features. First, the study addresses outcomes that are meaningful for patients and society, namely functional ability and well being. Second, it is targeted at a health problem that is likely to increase as the population ages, contributing to an area in which there are minimal rigorous intervention studies. If the programme is effective, it will provide a model that can be easily adapted and adopted by existing primary care or community services.

## Key messages

• 		Many observational studies and associated systematic reviews have demonstrated delayed physical and psychological recovery for survivors of a critical illness, often beyond 6 months after discharge.

• 		There have been limited interventional studies aimed at improving the recovery process for this patient cohort.

• 		This novel RCT with blinded assessment tests the effects of an eight-week, home-based, individually tailored physical rehabilitation programme on physical and psychological recovery for survivors of a critical illness after discharge from hospital.

• 		Participants in the intervention group receive graded, individualized endurance and strength training, focusing initially on lower limb exercises and walking, with warm up stretches, and progresses to the addition of core stabilization and upper limb exercises.

• 		Participant outcomes, including physical endurance, HRQOL, and psychological well being (depression, anxiety, stress, avoidance, intrusive thoughts) will be assessed using standard instruments at weeks 1, 8 and 26 after hospital discharge.

## Abbreviations

DASS = Depression, Anxiety, and Stress Scale; HRQOL = health-related quality of life; ICU = intensive care unit; IES = Impact of Events Scale; 6 MWT = 6 minute walk test; PF = Physical Functioning (subscale); RCT = randomized controlled trial; 8 RM = eight-repetition maximum; SF-36 = Short-Form-36 version 2.

## Competing interests

The authors declare that they have no competing interests.

## Authors' contributions

All authors contributed to the study design and methods, and the development of the grant application that was the source for this paper. JA specifically contributed to development of the exercise manual. MK, SM and DE specifically contributed to the statistical methods and power calculations. DE drafted the manuscript and all other authors critically revised it for important intellectual content. All authors approved the final version of the manuscript for publication.

## Figures and Tables

**Table 1:** Participant activities and study contact

Participant activities	Home visits after discharge (weeks)^a^				
	
	1 (baseline)	3	6	8 (short term)	26 (long term)
Outcome assessment: control and intervention groups					
Health-related quality of life (SF-36)	Visit 1	-	-	Visit 5	Visit 6
Physical endurance (6 MWT/Borg)	Visit 1	-	-	Visit 5	Visit 6
Psychological status (DASS/IES)	Visit 1	-	-	Visit 5	Visit 6

Intervention group					
Strength training	Visit 2	Visit 3	Visit 4	-	-
Endurance training	Visit 2	Visit 3	Visit 4	-	-

^a ^Visits 1, 5 and 6 are conducted by an assessor blinded to the study group; visits 2, 3 and 4 are conducted by a 'trainer' not involved in assessment, with follow up phone calls during weeks 2, 3, 5, 7 and 8 of the programme.

Borg, modified Borg Scale; DASS, Depression, Anxiety, and Stress Scale; IES, Impact of Events Scale; 6 MWT, 6 min walk test; SF-36, Short-Form-36 version 2.
